# Workforce Review of Health Information Technology Associate-Degree Graduates: 2017-2024

**DOI:** 10.63116/AH.000000002

**Published:** 2026-06-30

**Authors:** Debra Hamada, Karima Lalani, Melanie Brodnik, Susan H. Fenton, Angela Kennedy, David T. Marc, Velma L. Payne, Rebecca Reynolds

**Affiliations:** 1 Loma Linda University; 2 University of Washington; 3 The Ohio State University; 4 The University of Texas Health Science Center at Houston; 5 Commission on Accreditation for Health Informatics and Information Management Education (CAHIIM); 6 The College of St. Scholastica; 7 The University of Arizona; 8 University of Tennessee Health Science Center

**Keywords:** career mobility, experiential learning, health information management, health information technology, mixed-methods research, professional competencies, RHIT credential, workforce entry

## Abstract

**Background:**

The career path of associate-degree health information technology (HIT) graduates continues to change quickly as quality and payment regulations, technology requirements, and workforce expectations evolve to meet the demands of the changing health care environment. Although HIT professionals play an essential role in accuracy, regulatory compliance, and the overall function of health information systems, there is limited research describing HIT workforce trends.

**Methods:**

A mixed-methods approach was used with 587 survey responses. Quantitative data were standardized by job category, including a newly developed “health information management (HIM) operations” group, and career movement was visualized using a Sankey diagram. Qualitative comments were analyzed using inductive thematic coding, intercoder reliability checks, and a reconciled codebook. Both data sources were combined to provide a clearer picture of workforce patterns and graduate experiences.

**Results:**

Graduates reported a wide range of initial positions after graduation, most commonly in medical coding, HIM operations, billing, and patient registration. Many described strong upward mobility, moving into supervisory HIM roles, revenue cycle leadership, and growing opportunities in data analytics. Internships were frequently linked to job placement, and remote or hybrid work remained common. Three themes emerged from the qualitative analysis: (1) degree outcomes—graduates found the degree practical and career-building; (2) credential value—the Registered Health Information Technician (RHIT) continues to hold value but is not consistently recognized by all employers; and (3) employment issues—experience requirements and wages remain ongoing challenges.

**Conclusions:**

Overall, HIT graduates see clear value in their education and report meaningful career growth. At the same time, familiar challenges persist in work experience requirements that limit job availability for HIT graduates. Strengthening experiential learning, improving communication with employers about HIT competencies, and creating more intentional academic-employer partnerships may help close these gaps. These insights are intended to support educators, employers, and the broader HIM community in developing more effective workforce strategies.

## Background

Health information technology (HIT) as a professional degree is offered at the associate-degree level with a standardized curriculum and specialty program accreditation through the Commission on Accreditation for Health Informatics and Information Management Education (CAHIIM). These graduates work in hospitals and other health care settings to ensure the accuracy of health records and specialize in medical coding of diagnoses and procedures for reimbursement and other uses. Many external forces, including regulations from the Centers for Medicare & Medicaid Services (CMS), requirements from the Office of the National Coordinator for Health Information Technology (ONC), and other state and federal initiatives, continue to drive change in the health information profession.^1^

Although there has been a call for expanding the HIT workforce from the ONC and others, most previous workforce analyses for health information management (HIM) have been conducted at the baccalaureate or master’s level.^2^^,^^3^ This study aims to provide a comprehensive picture of how these changes are impacting initial and subsequent job attainment for HIT graduates. Graduates of CAHIIM-accredited associate HIT programs between 2017 and 2024 were surveyed (see appendix for the complete survey instrument). Respondents provided data on acquired jobs since graduation, including job category, job level, start and end dates, job location, state of employment, and job mode. The overarching goal was to collect data to provide stakeholders with a clear picture of employment trends, catalytic movements, and workforce challenges. In addition, identified employment trends will enable informed analysis of employer-valued skills, which in turn will influence training and educational efforts within the domains.

There is a noticeable lack of academic studies that directly address this specific focus; however, a small number of tangentially aligned studies offer relevant insights and warrant brief discussion. Through case study research, Broughton and Kortegast^4^ posited that attaining an associate’s degree, specifically the AS in HIT, was associated with favorable employment outcomes after graduation. Although the current study’s findings are mixed, they support these earlier results. In addition, the cross-sectional study conducted by Fenton et al^3^ demonstrated that the Registered Health Information Technician (RHIT) credential was the most frequently required among HIM online job postings. Moreover, this cross-sectional study concluded that “most HIM jobs require an associate degree or above.” This study further found that HIT job postings demonstrated considerable variability in job requirements and in the types of jobs held by HIM professionals. Similar findings from Madlock-Brown, Sharp, and Reynolds reinforce that most HIM jobs require a minimum of an associate degree, coding proficiency, and the RHIT credential. Gürcan^5^ echoed these findings,^6^ underscoring the necessity of certification and the importance of experience in job posting requirements.

Although this study was domestically focused, comparisons with Marc et al’s global workforce study and its findings are essential for understanding the prevalence of data analytics positions.^7^ This global workforce study concluded that the United States lagged behind the United Kingdom, Canada, Australia, and India in the prevalence of data analytics jobs. This study clearly demonstrates that, 5 years later, data analytics employment opportunities are growing domestically, thereby lessening the differences identified by Marc et al. In addition, Haried et al^8^ and Gjorgioski et al^9^ highlighted the prominence of data analytics skills as a desired qualification in job advertisements.

The Bureau of Labor Statistics projects a 15% increase in workforce demand for health information technologists and medical registrars.^10^ It is essential to understand the job outlook and career progression to recruit the HIM workforce needed to meet the health care industry’s needs. Considering these workforce projections, it is critical to examine, through research, the entry-level skill sets currently being used. This study offers insight into both the initial and subsequent job categories reported by survey respondents.

## Methods

A mixed-methods approach was used for this study. The combination of quantitative and qualitative approaches enabled a comprehensive, in-depth exploration of the research goal of illuminating current workforce trends among HIT associate-degree graduates. Data were exported from the survey tool into Excel for analysis. Following review and approval by the CAHIIM Board of Directors, participants were informed of the study’s purpose, and consent was implied through voluntary participation; participants were also advised that they could decline or withdraw at any time without penalty. The workforce survey was distributed to 227 accredited programs. Program directors were asked to disseminate the survey to eligible graduates. Emails were sent twice in October and November 2024, requesting that programs share the survey with graduates. Recruitment materials included participant incentives, including entry into a drawing for 1 of 10 registrations to the Summit on Higher Education and multiple $50 gift cards. The team of experienced researchers developed the survey instrument’s categories after reviewing AHIMA curricular domains and the current AHIMA Career Map.^11^^,^^12^

### Quantitative Data Collection

Respondents were permitted to report up to 5 distinct job positions held since completing a HIT program. Job records were first cleaned and standardized to ensure consistency across free-text responses. This process included converting titles to lowercase, removing leading modifiers such as “other-,” collapsing whitespace, and eliminating entries that were blank, whitespace-only, or represented noninformative placeholders (eg, “NA,” “none”). For each respondent, job entries were chronologically ordered, and the first reported job was identified as the individual’ initial postgraduation role. The final job listed was treated as the respondent’s most recent (current) role. For respondents who reported only 1 job, the same position was coded as both first and current.

A descriptive job-transition visualization was developed using a Sankey diagram to characterize career mobility within the health information workforce. The diagram displays the distribution of graduates’ first reported positions on the left axis and their current reported positions on the right axis, with flows between nodes representing the number of respondents transitioning between specific job categories. To enhance interpretability and reduce visual noise, job categories with fewer than 10 total observations across first and current positions were excluded from the visualization.

All quantitative data management, cleaning, and visualization procedures were conducted using R (version 4.4.1). Key packages included *Tidyverse* for data wrangling, *Lubridate* for date handling, *Ggalluvial* for generating the alluvial/Sankey diagram, and *Ggfittext* to optimize label placement within diagram strata.

As part of the data validation process, the participant’s job category assignment was conducted through a structured review process. Initially, 2 independent reviewers examined each assigned job category against the job categories provided in the data collection instrument to determine whether the participant's job category appeared accurate or was missing. After the initial review, a third reviewer examined cases where the 2 initial reviewers disagreed on the job category assignment. The third reviewer assessed the assignment to resolve the discrepancy and determine the final job category. In situations where the available information did not support a single category, additional follow-up was conducted through multiple rounds of communication among all reviewers to gather clarifying details. This ensured that each job was assigned to the most appropriate job category.

Table 1 presents the job categories generated by the researchers. It is important to note that the job categories on the original data collection instrument did not include a general HIM category, which was an oversight. Because of this omission, it appears that many participants selected the release of information (ROI) job category when the HIM job category would have been a more appropriate assignment. Thus, to address this gap and to avoid assigning cases to the ROI category when it was not the primary function, a general HIM operations category was created and applied as needed during the review process.

**Table 1.** Researcher RHIT Job Categories Used for Classification

**Category**	**Category**
Coding	Consumer health information and engagement
Clinical documentation integrity	Data, information, and analytics (business intelligence)
Education and training	Electronic health record solutions and support (including patient portals)
HIM Leadership and Supervision	Institutional compliance
Quality and performance improvement	Release of information
Revenue cycle management	Other (please specify)
HIM operations	

Abbreviations: HIM, health information management; RHIT, Registered Health Information Technician.

### Qualitative Data Collection

All respondents’ open-ended survey responses were exported from the survey platform into an Excel dataset for analysis. Two researchers employed an inductive thematic approach, allowing themes to emerge directly from the data rather than applying a predetermined framework. The coding process began with a line-by-line review of all responses and the development of an initial codebook that included provisional codes, working definitions, and illustrative excerpts. A second researcher reviewed this draft codebook, and both researchers independently coded the entire dataset using the preliminary structure.

Following independent coding, intercoder reliability was assessed to evaluate consistency in code application. The researchers negotiated agreements through virtual meetings to reconcile discrepancies, refine code boundaries, and clarify definitions, resulting in a final consensus-based codebook. This refined codebook was subsequently applied to the entire dataset to ensure alignment and analytical accuracy. Table 2 presents the final set of inductive codes used to identify the study’s emergent themes.

**Table 2.** Inductive Codes—Dominant Category

Dominant category
Career
Continued education
COVID-19
Credential
Degree format
Degree outcome
Degree value
Education
Employment
School/Employer collaboration
Skill deficit
Skill enhancement
Student expectation

## Results

### Quantitative Results

A total of 589 individuals responded to the survey. Two responses were removed due to incomplete data, resulting in a sample of 587 respondents. Across the sample, individuals reported between 1 and 5 jobs held since graduation, with respondents averaging 1.4 reported positions (SD = 0.76). Collectively, the dataset included 42 unique job titles and 871 reported total positions. The 10 most frequently reported job categories are presented in Table 3.

**Table 3.** Ten Most Reported Job Categories

**Job**	**Frequency**
Coding	248
Other—HIM operations	98
Other—billing and registration	83
HIM management/supervision	76
Revenue cycle management	46
Release of information	36
Other—service industry	35
Data/information/analytics (business intelligence)	30
Other—clinical	27
Education	22

Abbreviation: HIM, health information management.

Respondents represented a broad range of professional experiences. Most reported positions were classified as entry level (n = 401), followed by mid-level (n = 276), advanced (n = 148), and expert roles (n = 46). Employers spanned multiple sectors, most commonly hospitals (n = 418), corporate health systems (n = 93), nonacute care facilities (eg, rehabilitation, long-term care, ambulatory settings; n = 58), government agencies (n = 46), vendors (eg, electronic health record, medical device, insurance companies; n = 42), academic institutions (n = 18), consulting firms (n = 12), and self-employed individuals (n = 8). Respondents reported jobs located across 43 US states; 8 states were not represented (Delaware, Maryland, Montana, New Hampshire, Rhode Island, South Dakota, Vermont, and Wyoming). Four respondents reported international employment.

Work modality varied across roles. A majority of the reported positions were classified as fully in-person (n = 418), followed by remote (n = 325), and hybrid arrangements (n = 128). Length of time in a position ranged from 0 to 6 years, with the following distribution: 0 years (n = 46), 1 year (n = 106), 2 years (n = 67), 3 years (n = 41), 4 years (n = 22), 5 years (n = 16), and 6 years (n = 12).

Figure 1 presents a Sankey diagram depicting the progression of HIT graduates from their first position following program completion to their most recent reported job role. To improve interpretability, only job categories with more than 10 observations were retained, and job titles were cleaned and standardized before analysis.

**Figure 1. Sankey diagram: Illustrating the Job Flow From the First Job to the Current Job. EHR, electronic health record; HIM, health information management.**
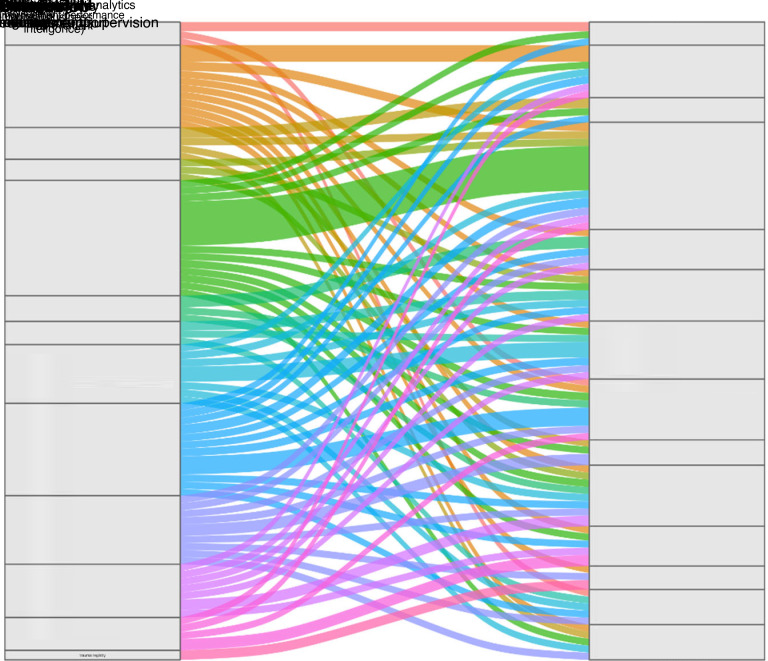


Graduates entered the workforce across a diverse set of initial roles, most commonly in medical coding, clinical positions, billing and registration, HIM management/supervision, HIM operations, data/information/analytics, and revenue cycle management. These entry-level roles form the left axis of the diagram. The right axis displays graduates’ current reported positions, reflecting subsequent career development and transitions across the health information domain.

The flows between the 2 axes illustrate the volume and direction of career transitions. Several notable patterns emerged. First, a substantial proportion of graduates remained within the same job category over time (eg, coding → coding), suggesting stability for individuals whose initial role aligned closely with their long-term career interests or specialized skill sets. Second, there was considerable upward and lateral mobility across the profession. For example, graduates who began coding, billing and registration, and HIM operations frequently transitioned into supervisory or advanced roles, such as HIM management/supervision, data/analytics, or revenue cycle management. These transitions indicate strong opportunities for career advancement within the health information workforce.

The diagram also highlights the role of data and analytics positions reported as a common career destination. Graduates transitioned into this area from a variety of first roles, including clinical, coding, revenue cycle, and HIM operations. This demonstrates the expanding relevance of data competencies and the versatility of HIT training in supporting analytics-oriented professional growth.

The Sankey visualization in Figure 1 underscores the heterogeneous and dynamic nature of HIT career pathways. Although some graduates remain in their initial domain, many exhibit substantial cross-functional movement, reflecting the breadth of career opportunities available within the health information profession. These findings suggest that HIT graduates are well-positioned to adapt to evolving workforce demands and assume roles requiring advanced technical, analytical, or managerial competencies.

### Qualitative Results

The thematic analysis revealed 3 overarching themes: degree outcomes, including perceived value and respondent degree affinity; credential value and impact; and employment issues. A total of 173 respondents contributed comments to the qualitative dataset, providing sufficient depth and repetition across responses to achieve thematic saturation.

Generally, respondents viewed the HIT degree outcomes favorably, with responses coded into 3 subthemes: career (positive—22), degree value (promotion after graduation—7), and degree outcome (competitive and desirable candidate—62). Overall, over half of the respondents’ comments were categorized as favorable outcomes. Table 4 summarizes these inductive comments by category, using comments from 10 respondents. These comments provide insight into the expressed value of the degree outcomes.

**Table 4.** Samples of Respondents’ Comments on Degree Outcomes—Favorable Responses

Degree outcome/subtheme	Response
Degree outcome (competitive and desirable candidate)	“I was able to obtain employment with a highly respected employer as a Medicolegal Technician I.”
Degree outcome (competitive and desirable candidate)	“I’ve experienced that having your RHIT certification you have opportunities for more job opportunities with better pay and can gain more knowledge and experience. MANY JOBS now require that you at least have a RHIT. THE RHIT CERTIFICATION IS WORTH HAVING!!”
Degree outcome (competitive and desirable candidate)	“Then once I was in the organization, I was eligible for degree required positions.”
Degree outcome (competitive and desirable candidate)	“Best decision ever to go back to school in this field.”
Career (positive)	“I love my job!”
Career (positive)	“Finding a job in my fields was not hard after graduation.”
Degree value (promotion after graduation)	“I was in school when I started my employment here and got a role in leadership after graduating the program.”

Abbreviation: RHIT, Registered Health Information Technician.

Oppositional comments, although in the minority, were received from respondents: career (negative—6), degree value (degree does not matter to employers—13), and degree outcome (questionable value—13). Table 5 presents inductive oppositional comments organized by category from respondents.

**Table 5.** Samples of Respondents' Comments on Degree Outcomes—Unfavorable Responses

Degree outcome/subtheme	Response
Degree value (degree does not matter to employers)	“Nobody wants to hire you based on your degree.”
Degree value (degree does not matter to employers)	“Many employers did not recognize how HIM education played into qualifications.”
Degree value (degree does not matter to employers)	“I wasn’t able to find employment as a coder after graduation.”
Degree outcome (questionable value)	“There is a severe disconnect between employers and colleges for what they are looking for.”
Degree outcome (questionable value)	“Not worth the schooling unless you plan on getting more credentials. There’s no appreciation for the field because it’s not clinical. It’s the ‘catch all’ and everything is the health information department’s problem to fix regardless of its registration, billing, insurance problem or even a patient complaint.”
Degree outcome (questionable value)	“I regret doing the HIT program at my college.”
Career (negative)	“Income levels have been lower than the average.”
Career (negative)	“I was completely mislead. The career rep was very unprofessional and impatient.”

Abbreviations: HIM, health information management; HIT, health information technology.

The second theme identified in the analysis, Credential Value and Impact, revealed a relatively balanced mix of positive and negative effects. Table 6 presents favorable comments coded as credential matter (18). Conversely, Table 7 also illustrates unfavorable opinions regarding credentials.

**Table 6.** Samples of Credential Value and Impact—Credentials Matter (Favorable) Responses

Response
“I’ve experienced that having your RHIT certification you have opportunities for more job opportunities with better pay and can gain more knowledge and experience. MANY JOBS now require that you at least have a RHIT. THE RHIT CERTIFICATION IS WORTH HAVING!!”
“RHIT was critical in the opportunities available to me postgraduation.”
“My decision to move forward with HIT and earn additional credentials has made a large difference in my overall HIM career.”
“I was hired as noncertified, after I passed my RHIT I got a raise.”
“I passed my RHIT exam shortly after graduating and began working shortly after that.”
“[T]he hospital paid for me to take AHIMA’s CHPS course and exam. I passed and now hold that certification as well and am transitioning to take over as the HIPAA Privacy Officer for the hospital.”
“I was able to get a job at a hospital immediately after getting my RHIT credentials. I took and passed the test quickly after graduation and that was when I was offered my position.”

Abbreviations: HIM, health information management; HIT, health information technology.

**Table 7.** Samples of Credential Value and Impact—Unfavorable Responses

Response
“Not worth the schooling unless you plan on getting more credentials. There’s no appreciation for the field because it’s not clinical. It’s the ‘catch all’ and everything is the health information departments problem to fix regardless of its a registration, billing, insurance problem or even a patient complaint.”
“I value the RHIT certification, worked hard to achieve it so continue to keep AHIMA membership and credential active. The health care company I work for does not recognize RHIT as a benefit for any position in HIM department. I feel there needs to be more education for health care companies to understand the credential and the knowledge an employee with an RHIT can bring to the department!”
“I was under the impression that the RHIT would blanket over the coding certificates and most interviews really did not view it that way.”
“Highly suggest getting CPC before RHIT.”
“Get all the certifications you can handle.”
“Even with RHIT credentials was finding it hard to find entry-level medical coding jobs.”
“Even with the credentials I had, I was unable to find anything that paid more than $16/hour.”“Many jobs wanted bachelor’s degrees, regardless of certification.”

Abbreviation: RHIT, Registered Health Information Technician.

The third theme, Employment, elicited a wide range of comments with varying meanings. Several subthemes were assigned to provide representative insight into RHIT graduates’ employment feelings: experience needed (49), low wages (21), internship led to job (16), remain in current position (15), entry-level job taken for experience (12), seeking remote work (8), left health care (6), and coding (5). Table 8 illustrates the meaning of the associated subthemes and indicator responses.

**Table 8.** Samples of Employment Issues Responses

**Subtheme**	**Response**
Experience needed	“Most people require that you have experience.”
Experience needed	“I really wish the program would have stressed being in an entry-level health care position would have been a positive because no one wants someone with no background even with holding an RHIT.”
Experience needed	“[H]ave not been selected for any due to a lack of experience in the particular positions.”
Experience needed	“Disappointing how long it took to find work in the field with no professional coding experience.”
Experience needed	“Tried to get a job along my education but every employer is asking for experience.”
Experience needed	“Utterly impossible to find a job postgraduation with RHIT and no experience.”
Low wages	“Pay is very disappointing.”
Low wages	“Employers requiring degree in the immediate area of the school are offering wages barely above minimum wage requirements.”
Internship led to job	“I was offered a position with the hospital I did my internship.”
Internship led to job	“I was hired by the hospital that I completed my practicum at I completed my practicum at.”
Remain in current position	“I was already working in the industry.”
Remain in current position	“I was unable to get a job in health information technology so I continued to do nursing.”
Entry-level job taken for experience	“[T]ake the entry level jobs to get your experience.”
Entry-level job taken for experience	“It was hard to find a job that paid well in the beginning, I had to obtain a position that did not require a degree at first. Then once I was in the organization, I was eligible for degree required positions. Thankfully I was only in the entry level position for 6 months.”
Seeking remote work	“Been very difficult to find good paying jobs especially remote ones.”
Seeking remote work	“Very hard to get into the remote.”
Left health care	“I went back to restaurant work because I could make more money and work less hours.”
Coding	“Unable to get a job in coding without experience. Very disappointing.”
Coding	“I love my job as a coder!”

Abbreviation: RHIT, Registered Health Information Technician.

## Discussion

This study provides a comprehensive picture of the current job market for associate-level HIT program graduates. As presented, overall quantitative findings aligned with prior findings.^3^^,^^6^^,^^7^ The survey results point to a wide range of job titles and career paths, with more than 40 unique roles reported across 43 states. Likewise, responses demonstrated a mixture of workplace settings and work modality. Although most individuals began their careers in entry-level positions, mid- to higher-level positions were identified as having clear advancement pathways, as shown. Prominent roles in data and analytics align with past research findings highlighting the desired skill. Of interest, Raghupathi and Raghupathi^13^ forecasted the impact of data analytics in health care in 2014.

Qualitative analysis found that HIT graduates have a favorable outlook on degree outcomes, with 91 favorable responses compared with 30 unfavorable responses. Favorable responses reflected an optimistic view that the degree and/or certification positioned them as desirable and competitive job candidates. Overall sentiments conveyed satisfaction with marketplace positioning and career advancement.

As in the first theme, respondents highlight a positive career trajectory following certification, indicating a positive influence on career advancement and earnings. However, unlike the first theme, there was more equity between favorable and unfavorable perspectives on credential value from the industry. Unfavorable sentiments focused prominently on the cost of the credential exam, misalignment with demonstrated competency in medical coding, and lack of recognition and awareness from employers of the credential value; Moede’s findings clearly support the lack of employer awareness of these programs and the RHIT certification.^14^

The subthematic coding of the third theme, employment, reveals various perspectives from respondents. Notably, 28% of respondents expressed concern about employers prioritizing significant work experience over credentials or degrees. In contrast, only 9% commented on the instrumental value of internships for securing jobs. In addition, many respondents raised concerns about low wages, with some citing this as a reason for leaving the health care industry; however, the responses did not identify any specific factors contributing to these low wages. This gap underscores the need for additional research to understand the contextual influences shaping salary trends in the HIT workforce. Although the number was not overwhelming, 8 individuals expressed frustration regarding the difficulty of obtaining remote work opportunities. Because only a small number of respondents mentioned challenges in finding remote work, it is not possible to conclude the reasons for these limited opportunities. Possible explanations include employers implementing return-to-office policies to support productivity, collaboration, and communication, as noted in recent workforce reports.^15^^,^^16^ It is also common for new entrants to start in on-site roles until they have demonstrated competency and can work independently.

The mixed perspectives from the qualitative analysis on how employers value the HIT degree and RHIT credential are noticeable and somewhat puzzling. These responses align with the 2021 Hanover Research Report, which found that although most employers continue to express strong confidence in higher education and believe a college degree is worthwhile, many still feel that graduates are not fully prepared for workforce demands.^17^ This disconnect echoes respondents’ comments about the importance of internships and prior experience. Together, these findings point to an ongoing need for academic programs to strengthen experiential learning and better align preparation with industry expectations.

### Strengths and Limitations

The mixed-methods approach employed in this study provides both strengths and uniqueness. Although quantitative literature exists for comparison, qualitative research is scarce. Qualitative findings enabled the accounting of respondents’ first-hand opinions. The participation of several project researchers provided a broad range of expertise and critical thinking. In addition, obtaining broad geographic and employment-site representation strengthens the findings. The use of participant data yielded strong quantitative and open-ended responses, providing direct insight into current workforce expectations and thereby informing the academic community.

The researchers relied on CAHIIM-accredited program directors to share the instrument with recent graduates, thereby challenging the widespread, holistic distribution. Because the survey was distributed via program directors and the total number of eligible graduates is unknown, a response rate could not be calculated. Graduates selected to participate in the survey, which may have influenced the results based on varying opinions on those who elected to participate versus those who did not. Although the researchers took concerted steps to limit coding bias, some degree of bias may remain. Respondents’ innate misrepresentations because of self-bias are another limitation, as is the selection of the ROI job category. The researchers created an HIM operations job category during data cleaning to improve analytic clarity and reduce fragmentation across similar roles; however, this decision may have introduced unintended, unmeasured bias into the data.

## Conclusions

This study offers a comprehensive look at the employment experiences of HIT associate-degree graduates. This study also highlights both the promise and the challenges of early career HIT professionals and provides a foundation for future research aimed at improving readiness, retention, and long-term workforce development. The findings confirm wide career diversity, clear advancement pathways, and strong opportunities in areas such as coding, HIM operations, revenue cycle, and the growing analytics space. Graduates overwhelmingly viewed their degree and credentials as valuable, though mixed perceptions, particularly around employer recognition, wage structures, and experience expectations, underscore ongoing gaps between academic preparation and workplace realities.

Experience emerged as a consistent barrier, whereas internships proved an essential but unevenly accessible advantage for some respondents. Concerns around compensation and limited remote opportunities also point to broader workforce dynamics that warrant further study. These insights reinforce the importance of strengthening experiential learning, deepening employer partnerships, and aligning curricula with the competencies most associated with career mobility. Future academic work should explore the role of employer partnerships and internships in closing the prior work experience gaps that new graduates may face entering the workforce.

## Disclosures

The authors have nothing to disclose.

## Funding

The authors received no funding for this research.

CE Quiz

## References

[1] Beesley K, McLeod A, Hewitt B, Moczygemba J. Health information management reimagined: assessing current professional skills and industry demand. Perspect Health Inf Manag. 2021;18(Winter):1b. Winter. PMC7883363 33633512

[2] Flite CA, Foster S, Houser SH, Essential skill and knowledge required for health data professionals: acontent analysis of job advertisements. Perspect Health Inf Manag. Summer. 2024;21(2):1c. PMC11605378 40134896

[3] Fenton SH, Marc DT, Kennedy A, Aligning the American Health Information Management Association entry-level curricula competencies and career map with industry job postings: cross-sectional study. JMIR Med Educ. 2022;8(3):e38004. Jul 7. doi:10.2196/38004. 35584188 PMC9305438

[4] Broughton TL, Kortegast C. A. I only see positive for those with a degree. J Appl Res Community Coll. 2024;31(2):137-151.

[5] Gürcan F. Identification of expertise roles and skill sets required for careers in health information management. Journal of Health Sciences. 2023;12(2):377-385.

[6] Madlock-Brown CR, Sharp MY, Reynolds RB. Assessing the prevalence of AHIMA-identified health informatics and information management careers and related skills: a cross-sectional study. Perspect Health Inf Manag. 2021;18(Spring):1k. Spring. PMC8120672 34035792

[7] Marc D, Butler-Henderson K, Dua P, Lalani K, Fenton SH. Global workforce trends in health informatics & information management. IOS Press; 1273–1277. doi:10.3233/SHTI190431. 31438130

[8] Haried P, Han Y, Annino D. A review of health information management and technology careers: a content-analysis of job advertisements. Journal of Business & Behavioral Sciences. 2021;33(1).

[9] Gjorgioski S, Riley M, Lee J, Workforce survey of Australian Health Information Management Graduates, 2017–2021: a 5-year follow-on study. Health Inf Manag. 2025;54(1):43-54. doi:10.1177/18333583231197936. 37753774 PMC11705756

[10] Statistics USBoL Occupational outlook for medical and health services managers. 2025; https://www.bls.gov/ooh/healthcare/health-information-technologists-and-medical-registrars.htm.

[11] Map AC AHIMA career map. https://my.ahima.org/career-mapping/career-map/.

[12] Domains AHIaIMH AHIMA health informatics and information management (HIIM) domains. https://www.nyhima.org/ahima-domains.

[13] Raghupathi W, Raghupathi V. Big data analytics in healthcare: promise and potential. Health Inf Sci Syst. 2014;2(1):3. doi:10.1186/2047-2501-2-3. 25825667 PMC4341817

[14] Moede A. Perceptions of northeastern Wisconsin health insurance employers toward hiring applicants with a health information technology two-year degree. 2023.

[15] Coacci J. Manhattan's offices are on track to be just as busy as pre-pandemic years as Wall Street and tech companies drag workers back to the office. Fortune.com. 2025; N.PAG–N.PAG.

[16] Cascio WF. The dynamics and complexities of return-to-office policies. Hum Resour Manage. 2026;65(2):591-604. doi:10.1002/hrm.70041.

[17] Finley A. How college contributes” to” workforce success: Employer views on what matters most. 2021.

